# Expression of Concern for Huang et al., “Complete Genome Sequence of Campylobacter coli Bacteriophage CAM-P21”

**DOI:** 10.1128/mra.00817-22

**Published:** 2022-09-07

**Authors:** American Society for Microbiology

**Affiliations:** Washington, DC, USA

## ANNOUNCEMENT

The American Society for Microbiology (ASM) and *Microbiology Resource Announcements* (MRA) are issuing this Expression of Concern regarding the following publication:

Huang H-H, Zhang Y, Asoshima N, Duc HM, Sato J, Masuda Y, Honjoh K-I, Miyamoto T. 2021. Complete genome sequence of Campylobacter coli bacteriophage CAM-P21. Microbiol Resour Announc 10:e00223-21. https://doi.org/10.1128/MRA.00223-21.

MRA was notified by a reader that sequence data reported in this paper are potentially inaccurate/incomplete. The concerns regarding Campylobacter phage CAM-P21 with GenBank accession number MW462221 are as follows:

1. The sequence does not include the three universal features of myoviruses: capsid biosynthetic proteins, packaging proteins (i.e., terminases), and lysis proteins.
2. HHpred analysis of the sequence showed no evidence that the protein submitted under NCBI accession number QRW43453 is a virion morphogenesis protein, as stated in the paper.

ASM sent an inquiry to the authors and requested a response along with the original, underlying data regarding these concerns. ASM has not received a response addressing the concerns with the published data. This Expression of Concern is issued pending the authors’ response and will be updated accordingly thereafter. If MRA and ASM do not receive the complete sequencing data and a thorough explanation to the above-mentioned concerns within 6 months, then ASM will proceed with a Publisher’s Retraction of this paper.

